# Faculty Members’ Perception, Implementation, and Challenges of Formative Assessment in Undergraduate Medical Education: A Cross-Sectional Study

**DOI:** 10.7759/cureus.74107

**Published:** 2024-11-20

**Authors:** Ali M AlAskari, Abdulmohsen H Al Elq, Eman R Mohamed, Manahel A Almulhem, Mohammad Zeeshan, Komail A Alabbad, Ali H AlSairafi, Abdulaziz M Alsharif

**Affiliations:** 1 Department of Otolaryngology - Head and Neck Surgery, Al Jabr Eye and ENT Hospital, Al Mubarraz, SAU; 2 Department of Medical Education, Imam Abdulrahman Bin Faisal University, Dammam, SAU; 3 Department of Medical Education, College of Medicine, Imam Abdulrahman Bin Faisal University, Dammam, SAU; 4 Department of Anesthesiology, King Fahad Hospital, Al Hofuf, SAU; 5 Department of Internal Medicine, Qatif Central Hospital, Al Qatif, SAU; 6 Department of Internal Medicine, Prince Saud Bin Jalawi Hospital, Al Mubarraz, SAU

**Keywords:** faculty members, formative assessment, medical curriculum, medical education, perception

## Abstract

Background: Medical curriculum reform requires quality assurance in all curricular activities including assessment. Such reform necessitates a shift of focus from summative assessment (SA) to formative assessment (FA). However, the implementation of FA confronts several challenges that hinder its proper application, especially with the increased number of students and the diversity of activities required from faculty members. Therefore, this study aims to explore the perception, implementation, and challenges facing faculty members during the application of FA to undergraduate medical students.

Methods: This is a cross-sectional study using an online survey to collect data from the pre-clinical and clinical faculty members of the College of Medicine (CoM) at Imam Abdulrahman Bin Faisal University (IAU), Dammam, Saudi Arabia. The survey consisted of two sections. The first section focused on the demographic and academic-related data, while the second section consisted of a validated self-administered questionnaire related to FA. The questionnaire comprised six domains regarding the level of perception, implementation, and challenges facing FA applications.

Results: Among the 347 faculty members who received the research survey, 132 members participated in this study (38%). Regarding awareness, 68.9% and 67.4% of the faculty members were aware of two subdomains, namely, "functions of formative evaluation and understanding of its usefulness" and "sharing and addressing educational attention and success criteria." However, with respect to the application of FA, only one-third of the members implemented the FA into their teaching process. Regarding barriers to applying FA, the majority of the participants agreed on the major barriers, which included required activities from the instructors (93.2%), the number of students (87.1%), and students’ engagement (84.1%).

Conclusions: There is a fair overall awareness regarding the concept and importance of FA. Despite that, the application of such an assessment method was limited. This may be attributed to several challenges, including a large number of students and the required activities from the faculty. Further studies are recommended to investigate the implementation of an FA feedback system and its impact on the learning process in the CoM at IAU.

## Introduction

The educational field has been modernized with new educational aims and goals. Therefore, new curricular standards were developed to accommodate the cultural, informational, and technological needs, as well as the learners’ evolving world. An example of the new standards is the evolution of new methods of assessment, including formative assessment (FA). Such assessment methods should be considered and applied to the curriculum, as well as the summative assessment (SA). SA aims to make a decision based on the learner’s performance such as admission, progression, or qualification exams [1]. On the other hand, FA is known as assessment for learning where it aims to provide the learners an insight into their performance in order to shape their competence before or during the learning process [1-3]. Feedback is the cornerstone in FA that provides the learners an insight to improve their performance. Moore described FA as continuous feedback and guidance provided throughout courses [4,5]. FA was first introduced in the early 1970s by Benjamin Bloom and has been considered a fundamental concept in the medical education field [6].

FA impacts the educational process of both the learners and the teachers positively as well as negatively. Regarding the teachers, it provides them with the information they need to analyze current learners' competence before and during class [7]. Therefore, it assists the teachers in identifying learners with deficiencies in the early stage, encourages teachers to apply various teaching methods, and promotes interactive strategies that result in the enhancement of their teaching skills [8]. On the student side, it assists them to understand how they are progressing toward achieving the objectives of the course to enhance their learning process [8,9]. Simultaneously, FA performance influences the learners’ performance in SA. The more FA the learners requested and participated in, the higher their SA performance was at the end of their learning experience [10]. Such a practice has a statistically significant impact on the results of SA and consequently on undergraduate medical education [11-16].

Recently, FA has been receiving global attention, specifically in developed countries as a guiding approach with the notable potential to improve teaching, as well as learning processes [14,15]. On the other hand, the developing countries including the Middle East region are lacking the application of FA, which is evident in the literature [15].

In the Kingdom of Saudi Arabia (KSA), the educational system is characterized by being teacher-centered in general where SA is the dominant assessment method [15]. However, FA started to gain more attention nowadays.

There has been a shift in the learners’ perception of assessment. Nowadays, they view the evaluation of learning as quality-oriented education, instead of examination-oriented education [17]. Several studies investigated the medical faculty and students’ perspectives regarding FA worldwide [18-20]. In the KSA, the perception of medical students and faculties regarding FA is minimally studied.

Therefore, this study aims to investigate the faculty members’ perception of FA, the extent of its application, and the challenges of its implementation in the College of Medicine (CoM), Imam Abdulrahman Bin Faisal University (IAU), Dammam, Saudi Arabia.

Research questions

What is the perception of the medical faculty members at IAU regarding FA in undergraduate medical education? To what extent do the medical faculty members at IAU implement FA in undergraduate medical education? How do the medical faculty members at IAU practice FA? What are the barriers that hinder the proper application of FA in undergraduate medical education from the perspective of the medical faculty members at IAU?

It is important to mention that this article was previously posted as a preprint to Research Square on December 6, 2022 (doi.org/10.21203/rs.3.rs-2285703/v1).

## Materials and methods

Study settings

This study was conducted at the CoM at IAU, which adopted the integrated problem-based learning curriculum for undergraduate medical students. A six-year Bachelor of Medicine, Bachelor of Surgery (MBBS) undergraduate program starts with two years of basic medical sciences followed by three years of clinical clerkship and ends with a mandatory one-year internship. The primary assessment method in both pre-clinical and clinical specialties is SA with some incorporation of FA in the curriculum.

Study design and participants

This is a descriptive cross-sectional study that was conducted from December 2021 to April 2022. The study included all medical faculty and staff members who are responsible for assessing undergraduate medical students in the basic medical sciences years (second and third year) and the clinical years (fourth, fifth, and sixth year). A total of 347 members from different specialties were invited to participate in the study, including 65 from basic medical science departments and 282 from clinical departments.

Sample size

Population size (N): 347 (Total clinical faculty = 282, Total basic medical sciences faculty = 65). Percent of outcome factors in the population (p): 50%

Margin of error as (d): 7% and 1-α (Confidence level) = 95% and Z is the value from the standard normal distribution reflecting the confidence level that was used (Z = 1.96 for 95%)

Total sample size (n) = 126

The following formula was used from the Open-epi sample size calculator (https://www.openepi.com)

n = [Np(1-p)]/ [(d2/Z21-α/2*(N-1)+p*(1-p)]

Development and distribution of the data collection tool

An online self-administered structured questionnaire was used to collect the data (Appendix 1). The questionnaire consisted of two sections: The demographic and general characteristic data and the FA tool.

The demographic and general characteristic data included age, gender, nationality, years of experience, degree of qualification, specialty, etc. This section was developed after reviewing the related literature.

The FA tool was created according to the guidelines of the theoretical framework developed by William [7] that included six dimensions: functions of FA and understanding of its usefulness, sharing and addressing educational attention and success criteria, methods of performing the FA, the temporality of FA and guidance, instructors training, and obstacles to the application of FA. The tool consisted of a validated 37 items with a pre-determined internal consistency of Cronbach's α of 0.854. A single answer on a scale of measurement extending from 1 to 5, where 1 represented strongly disagree and 5 represented strongly agree [14].

The total possible score on the perception level has been categorized into three main categories: poor average (> 50), fair average (50 > 75), and good average (> 75).

Data were collected by an online survey written in English, which is the main teaching language in the CoM at IAU. The online survey was made using QuestionPro forms, which were provided by the university for free. A link hyperlink was sent through the official university email to all faculty/staff members who are responsible for teaching undergraduate students in basic medical sciences and clinical years. In our study, staff members are residents, specialists, or consultants, while faculty members are lecturers, assistant professors, associate professors, or professors. Filling in the questionnaire required approximately 5-10 minutes for each participant. In order to submit the questionnaire, the participant is required to answer all the questions as only a fully completed survey was accepted. Then, the data of the accepted questionnaire were exported directly into the connected Google Sheets, which was used for data analysis. The data were collected from the first of December 2021 until the 15th of February 2022.

Ethical considerations 

Prior to conducting the study, Institutional Review Board (IRB) approval has been obtained from IAU IRB with reference number (IRB-UGS-2021-01-352). A consent form was sent to all the participants in order to sign with prior knowledge of the objectives of this research. The participants were informed that their participation was voluntary, and they had the right to withdraw from the study at any point without any obligations. All gained information has been kept confidential.

Data analysis

Data were coded and tabulated, and statistical analysis was carried out using Statistical Product and Service Solutions (SPSS, version 23.0; IBM SPSS Statistics for Windows, Armonk, NY). Categorical variables were represented as numbers and percentages. Contentious variables were presented as mean and standard deviation. The chi-square test was used to establish an association between two categorical variables. A T-test was used to compare mean score values between two categories, while one-way ANOVA was to compare the mean scores between three categories. A p-value less than 0.05 was considered statistically significant, with a confidence interval of 95%.

## Results

Demographic and general characteristic data

A total of 132 out of 347 faculty and staff members participated in this study, with a response rate of 38%.

The mean age of the participants was 42.5, and the majority were female faculty members (56.1%). The participants had different academic experiences; faculty members (68.2%) were the most common, almost two-thirds (65.2%) had more than 10 years of experience, and the majority were responsible for teaching clinical years (90.9%) (Table 1).

**Table 1:** The demographic and general characteristic data of the participants

	Items	Frequencies	Percentages
Age (years)	Mean (±SD)	42.5 (±9.5)	
Gender	Male	58	43.9
	Female	74	56.1
Nationality	Saudi	88	66.7
	Non-Saudi	44	33.3
Fields	Biomedical	12	9.1
	Clinical	120	90.9
Degree of qualification	Faculty	90	68.2
	Staff	42	31.8
Years of experience	Less than 5	18	13.6
	5-10	28	21.2
	10 and more	86	65.2
Native language	Arabic	114	86.4
	English	7	5.3
	Others (Urdu, Hindi, French)	11	8.3

FA tool

In general, awareness of the FA domain was rated higher than its application domain. Regarding awareness, both subdomains were rated positively. The “functions of formative evaluation and understanding of its usefulness” subdomain (68.9%) was rated higher than the “sharing and addressing educational attentions and success criteria” subdomain (67.4%). On the other hand, the application of FA subdomains was rated lower. Less than two-thirds of the participants (60.6%) used various methods to apply FA with a dominance of classroom questioning (86.4%). As regards applying the temporality of formative evaluation and guidance, it demonstrates that only one-third of the participants (33.3%) applied the temporality of formative evaluation and guidance (Table 2).

**Table 2:** Overall mean score of the participants’ awareness and application of the formative assessment tool *5 = Strongly agree, 4 = Agree, 3 = Unsure, 2 = Disagree, 1 = Strongly disagree, ** Total domain score > 75%, Positive Responses = Strongly Agree + Agree

		Mean (±SD)*	Positive Responses (%)
Awareness	Functions of formative evaluation and understanding of its usefulness		
	Identifying the student's strengths and weaknesses	4.2 (±0.9)	114 (86.4%)
	Guiding student progress	4.19 (±0.82)	111 (84.1%)
	Increasing the student's autonomy	3.9 (±0.85)	96 (72.7%)
	Direct teaching plan	4.05 (±0.77)	106 (80.3%)
	Domain Mean	16.4 (±2.7)	
	Overall Awareness**	91 (68.9%)	
	Sharing and addressing educational attention and success criteria		
	I share the objectives of each course with my students	4.33 (±0.83)	115 (87.1%)
	I explain the learning goals to achieve with my students	4.38 (±0.85)	118 (89.4%)
	I discuss the criteria for success with my students	4.06 (±0.97)	101 (76.5%)
	I discuss the modalities of the summative assessment with my students	4.1 (±0.93)	101 (76.5%)
	I discuss the modalities of formative assessment with my students	3.97 (±0.96)	93 (70.5%)
	Domain Mean	20.8 (±3.8)	
	Overall Awareness**	89 (67.4%)	
Application	Methods of performing the formative evaluation		
	Classroom questioning	4.19 (±0.8)	114 (86.4%)
	Exercises and tests	4.06 (±0.84)	109 (82.6%)
	Group discussion	4.1 (±1)	106 (80.3%)
	Self-evaluation	3.9 (±0.89)	97 (73.5%)
	Peer evaluation	3.97 (±0.86)	101 (76.5%)
	Digital assessment	3.9 (±0.91)	92 (69.7%)
	Domain Mean	24.1 (±3.6)	
	Overall Awareness (Application)**	80 (60.6%)	
	Temporality of formative evaluation and guidance		
	I do the formative assessment after each teaching activity	3.54 (±1.01)	78 (59.1%)
	I do the formative assessment at the end of a session of the course	3.73 (±0.81)	90 (68.2%)
	I do the formative assessment at the end of a course	3.67 (±0.93)	88 (66.7%)
	I do the formative assessment before the summary evaluation of a course	3.41 (±0.96)	66 (50%)
	I give formative feedback to my students after the formative evaluation activities	3.81 (±0.89)	89 (67.4%)
	I give individual feedback after the activities of the formative evaluation	3.79 (±0.85)	92 (69.7%)
	I propose a regulation such as ‘remaking one or more sequences of teaching’	3.53 (±0.88)	65 (49.2%)
	I propose a regulation such as ‘change the teaching material’	3.52 (±0.9)	72 (54.5%)
	I propose a regulation such as ‘increase the hourly volume of a course’	3.52 (±0.94)	70 (53%)
	I propose a regulation such as ‘give more explanations’	3.8 (±0.86)	91 (68.9%)
	I propose a regulation such as ‘do additional exercises’	3.81 (±0.87)	91 (68.9%)
	Domain Mean	40.1 (±7)	
	Overall Awareness (Application)**	44 (33.3%)	

The majority of the participants (64.4%) have received training programs that include workshops, seminars, or webinars on applying FA with a significant difference (p<0.001).

Out of 132 respondents, a significantly higher number of respondents 81 (61.4%) were applying informal FA as compared to formal FA (p=0.0002).

Participants’ demographics and the FA tool (awareness, application, and barriers)

There were no statistically significant differences based on gender and age groups (Table 3).

**Table 3:** Relationship of sociodemographic data (gender and age) with all the domains of the formative assessment tool

Domains	Min-Max	Gender			Age (Years)		
		Male	Female	p-values	≤ 40	> 40	p-values
Functions of formative evaluation and understanding of its usefulness	4-20	16.6 (±2.7)	16.2 (±2.7)	0.445	16.3 (±2.7)	16.4 (±2.8)	0.83
Sharing and addressing educational attention and success criteria	5-25	21.1 (±3.6)	20.6 (±3.9)	0.454	20.8 (±4)	20.8 (±3.6)	0.988
Methods of performing the formative evaluation	6-30	24.4 (±3.2)	23.9 (±3.9)	0.416	24.2 (±4.1)	24 (±3.2)	0.817
The temporality of formative evaluation and guidance	11-55	39.5 (±6.3)	40.6 (±7.6)	0.386	40.4 (±7.6)	39.9 (±6.4)	0.678
Instructor’s training	6-30	24.2 (±3.5)	23.1 (±3.7)	0.076	24.1 (±3.6)	23.1 (±3.6)	0.147
Obstacles to the application of formative evaluation	5-25	19.4 (±2.9)	19.1 (±3.4)	0.7	19.4 (±3.3)	19.1 (±3)	0.554

Similarly, there were no significant differences based on position and years of experience with respect to both the awareness subdomains. However, a statistically significant difference was found between years of experience in relation to the application subdomains; methods of performing the formative evaluation (p=0.01) and temporality of formative evaluation and guidance (p=0.05). The participants with 5-10 years of experience rated both subdomains higher than other participants (25.4 (±3.2) and 42.2 (±6.8)), respectively. Additionally, a highly significant difference was found between the participants’ positions in relation to the temporality of formative evaluation and guidance subdomain (p=0.007). The staff rated this subdomain higher than the faculty (42.5 (±7.9) vs. 39 (±6.3)), respectively (Table 4).

**Table 4:** Relationship of academic career experience (position and years of experience) with all the domains of the formative assessment tool *Significantly high mean scores Staff = Residents, specialists, or consultants, Faculty = Lecturers, assistant professors, associate professors, or professors

Domains	Min-Max	Position			Experience (Years)			
		Faculty	Staff	p-values	< 5	5-0	> 10	p-values
Functions of formative evaluation and understanding of its usefulness	4-20	16.2 (±2.7)	16.7 (±2.6)	0.32	16.1 (±2.9)	16 (±1.9)	16.5 (±2.9)	0.6
Sharing and addressing educational attention and success criteria	5-25	20.7 (±3.9)	21.1 (±3.6)	0.6	19.8 (±4.5)	21.4 (±3)	20.9 (±3.8)	0.4
Methods of performing the formative evaluation	6-30	23.8 (±3.1)	24.9 (±4.6)	0.113	22.1 (±4.2)	25.4 (±3.2)*	24.1 (±3.5)	0.01
The temporality of formative evaluation and guidance	11-55	39 (±6.3)	42.5 (±7.9)*	0.007	37.1 (±6.7)	42.2 (±6.8)*	40.1 (±7)	0.05
Instructor’s training	6-30	24.1 (±3.3)	24.7 (±3.5)	0.4	23.1 (±3.7)	24 (±3.5)	23.6 (±3.7)	0.697
Obstacles to the application of formative evaluation	5-25	20.9 (±2.1)	21.5 (±2.3)	0.15	18.3 (±3)	19.6 (±3.4)	19.3 (±3.1)	0.396

**Figure 1:**
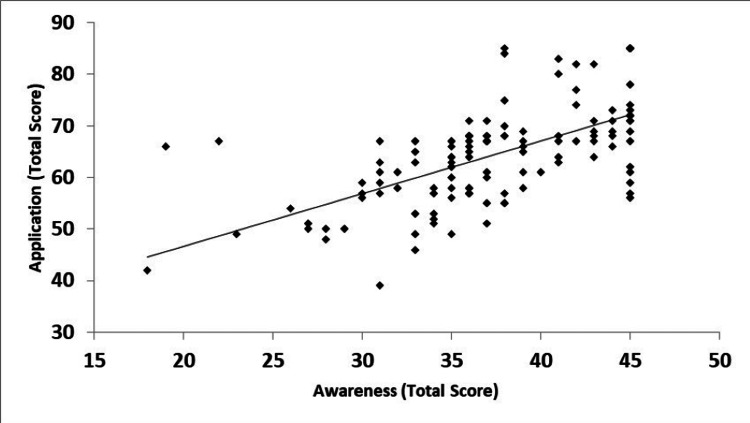
Relationship between awareness and application domains of the formative assessment scale Pearson correlation coefficient, r = 0.62*; p-<0.0001

Additionally, the current study revealed a significant positive correlation between the awareness toward FA, especially those that could be tackled inside the classroom, and its application. It showed that the more the participants were aware of FA, the more likely they were able to implement classroom activities, such as classroom questioning, exercises and tests, and group discussion (86.4%, 82.6%, and 80.3%, respectively) (Figure 1). 

**Figure 2:**
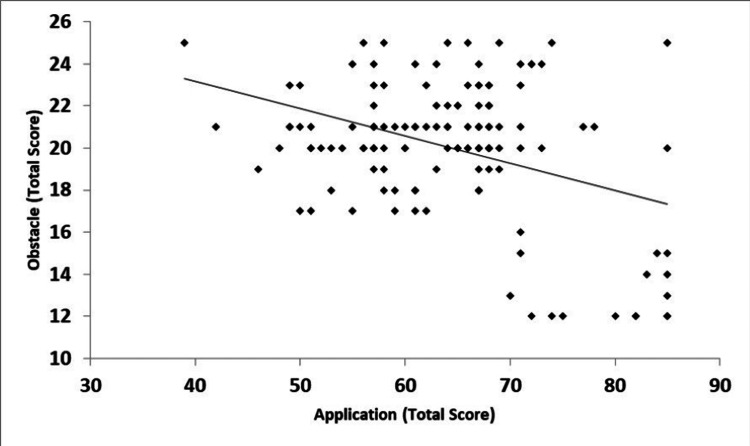
Relationship between application and obstacles domains of the formative assessment scale Pearson correlation coefficient, r = -0.38*; p-<0.0001

On the other hand, our study showed that the more the confronted barriers, the less the application of FA, especially such applications concerned with the temporality of formative evaluation and guidance (Figure 2).

Barriers to applying FA

**Figure 3:**
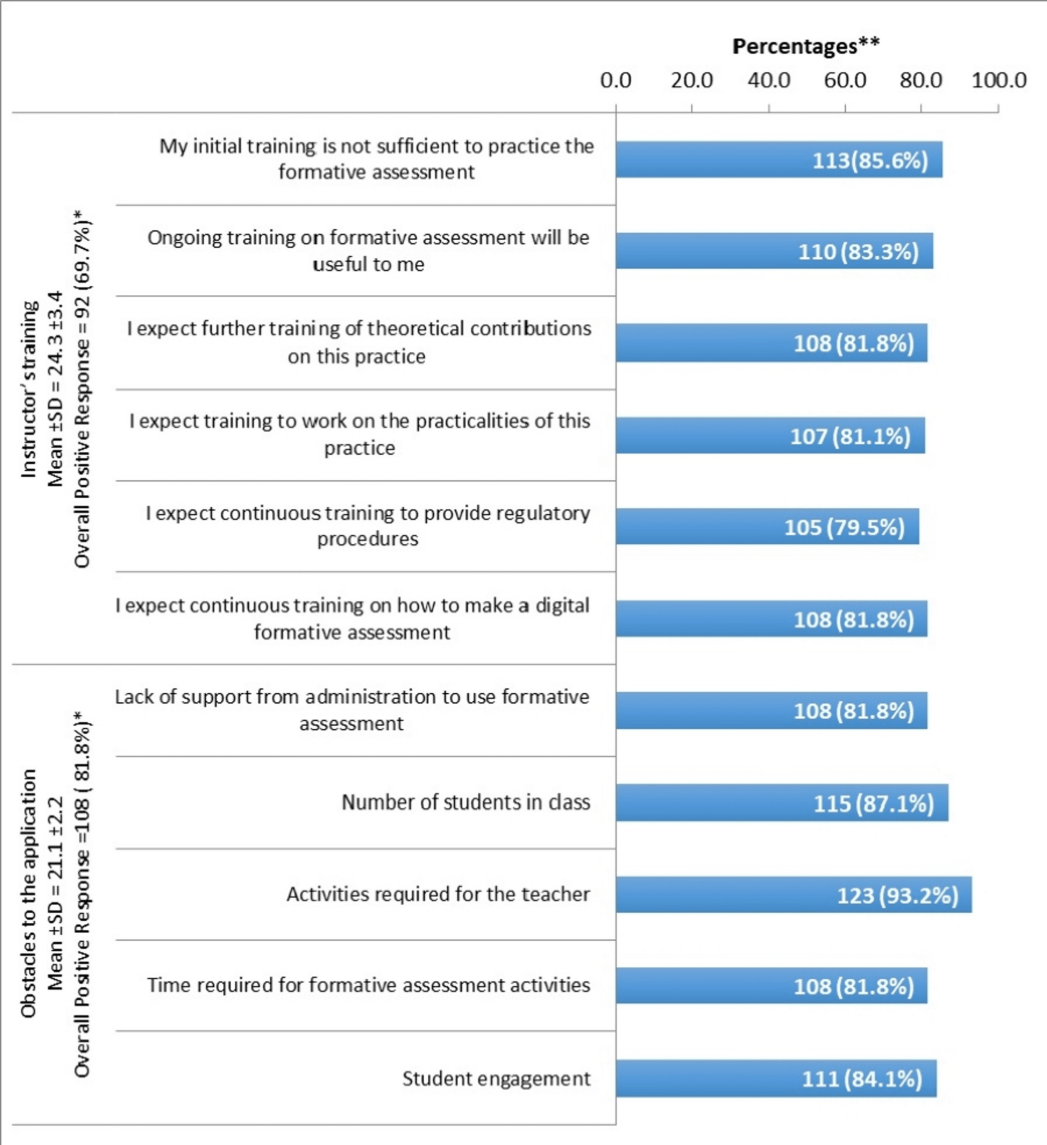
Barriers to applying the formative assessment Total domain score > 75%, Percentages of positive responses (Strongly agree + Agree)

In general, there was an overall positive response regarding the main two domains: instructor training (69.7%) and obstacles to the application (81.8%). With respect to training, the majority of the participants were aware of the lack and importance of the training sessions as they stated that their initial training was insufficient to apply FA (85.6%) and the majority (83.3%) agreed on the need for ongoing training sessions. Regarding the confronted obstacles, the majority of the participants agreed on the major barriers, which include required activities from the instructors (93.2%), the number of students (87.1%), and students’ engagement (84.1%) (Figure 3).

## Discussion

The transition from traditional to innovative curricula has accelerated recently, necessitating the use of a comprehensive assessment method such as FA to address the need for curricular reform [6]. However, such practice is difficult to apply due to time consumption, academic load, variant faculty understanding and ability, and poor administrative support [12,15,21]. Therefore, this study aimed to investigate the perception, application, and barriers that impede the implementation of FA among the faculty and staff of the CoM at IAU in the KSA.

Concerning the faculty perceptions of the FA, our study findings revealed a reasonable level of awareness (68.9%) of its functionality and usefulness (first domain) with good response in its two sub-domains termed "identifying students' strengths and weaknesses" and "directing the teaching plans" (86.4% and 80.3%, respectively). This finding was in line with another study that also emphasized the crucial role of FA in gaining faculty members' insight into their student's weaknesses and strengths and identifying where the curricular gaps exist [11,12]. This positive perception of FA was in agreement with other local Saudi studies [16,22].

Regarding the second domain concerning the awareness of FA named, sharing and addressing educational attention and success criteria, our study also revealed a good awareness score level of multiple sub-domains. These include sharing learning objectives (87.1%), discussing criteria of success (76.5%), and explaining the modalities of SA and FA (76.5% and 70.5%, respectively). This was consistent with another study stating that sharing learning goals, success criteria, and assessment criteria with students are essential attributes of effective FA [14]. Additionally, a considerable positive response was shown (89.4%) for explaining the learning goals to the students prior to the start of teaching sessions to achieve the desired learning outcomes. This significant level of awareness may reflect the familiarity of the faculty members regarding this type of assessment method as most of the study participants have a considerable number of years of teaching experience, which is consistent with the mean age of the participants. Such academic experience may enhance the faculty members’ ability to apply such assessment methods [13].

FA strategies range from informal classroom observations to formal curricular integrated techniques [14]. However, the informal FA strategies, in terms of classroom questioning, exercises and tests, group discussion, and digital assessment, take precedence over the formal formative strategies [13,23,24], which were in line with our study findings, in which approximately one-third of the participants (35.6%) applied both formal and informal strategies and the majority of the participants (61.4%) agreed on applying the informal techniques.

Despite that, FA in medical education should be a regular process rather than a series of single events [25,26]. The current study contrasted this assumption as only a third of the participants (33.3%) stuck to the temporality of FA evaluation and guidance. This finding may shed light on another factor that hinders the application of FA. This factor may be related to the various challenges the faculty or staff have which may impede them from applying such type of assessment.

Additionally, the current study revealed a significant positive correlation between the awareness toward FA, especially those that could be tackled inside the classroom, and its application. This is in agreement with another study that mentioned that the implementation of FA in classrooms is difficult due to the lack of teachers’ knowledge [13,16].

On the other hand, the barriers to applying FA hinder its application despite instructors’ awareness [16,27], which is consistent with our study. This may be attributed to the limited application of formal FA strategies in the current study. Such limited application of FA may be attributed to facing multiple barriers [13]. These include time consumption, additional effort, and further resources [12,13,20,24,28]. Additionally, the adopted assessment method worldwide, including the KSA, in undergraduate medical education is mostly SA [15,25,29]. Moreover, negative students’ perceptions of FA and poor commitment to its requirements hinder its practice [24,30].

Furthermore, the insufficient, general, and unfocused training of the instructors hinders the application of such assessment methods [13]. This finding is consistent with our result: despite receiving different forms of training (workshop/seminar/webinar) on applying FA, the faculty members and staff stated that the training was insufficient and signified their need for theoretical and practical continuous training, which was in agreement with another study [24].

Further barriers include a high academic workload on the instructors [15,24,31,32], a large number of students, and class crowdedness [27,33,34], as well as the lack of administrative follow-up and support [13]. In addition to time consumption and students’ engagement, these barriers are consistent with the findings of the current study.

With reference to the sociodemographic data of our participants, age and gender showed no significant relationship with the various FA tool domains. To illustrate, despite the superiority of the number of female participants, we found that it has no significant impact on FA tool domains. In contrast, Alotaibi noted that female teachers in the KSA have more significant positive attitudes towards FA [35]. This result may be attributed to the equal distribution of the workload burden among both male and female instructors [16,30,33].

Concerning the academic experience of the participants, our study found that more than two-thirds of participants (65.2%) have more than 10 years of teaching experience and the highest mean scores of FA application in terms of methods and temporality (25.4 (±3.2) and 42.2 (±6.8), respectively) in those who have 5-10 years of experience. In contrast, teachers who have less than five years of teaching experience scored the lowest (22.1 (±4.2) and 37.1 (±6.7), respectively). This finding may rationalize the reasonable level of FA application inside the classroom which is more approachable than the formal one.

Furthermore, our study revealed that staff has superiority over faculty regarding the FA temporality with mean scores (42.3 (±7.9) and 39.1 (±6.3), respectively). This result could be explained by the fact that staff members are not as devoted to a heavy teaching and administrative schedule as faculty members, allowing them to consume more time conducting FA.

This study faced multiple limitations related to filling out the survey and time constraints. The data collection period was during multiple academic vacations, which may have impacted the response rate (38%) of the current study. In addition to the response rate, the study was conducted in one academic institute, which may affect the generalizability of our result. These limitations hindered a wide range of the sample.

## Conclusions

The medical faculty and staff were fairly aware of FA with respect to its usefulness and function subdomains. However, the application domain showed a poor implementation of FA by faculty members in their teaching process specifically in the "temporality of formative evaluation and guidance" subdomain. Despite fairly aware faculty members of FA, there is a poor implementation that may be attributed to several barriers, including the large number of students and the increased workload.

Based on the findings of this study, it is recommended to conduct further studies related to the factors that decrease the awareness and application of FA in medical schools. In addition, educational institutes should establish continuous professional training workshops to equip medical faculty members with the skills to apply FA. Additionally, it is recommended to split medical students into several groups to decrease the load on faculty members, which would enable them to apply FA. Furthermore, implementing a mandatory FA feedback system would be beneficial to assist the students, faculty, and administrators in gaining the necessary dividends from FA to produce lifelong learner graduates. Finally, a protected time should be considered by the administrative personnel for both faculty as well as students.

## Appendices

Participation consent

This questionnaire (Tables 5-6) aims to identify the perception of medical faculty towards the application of formative assessment and obstacles preventing well-functioning and establishment of this tool at the College of Medicine, Imam Abdulrahman Bin Faisal University. We hope that you complete all the questions written in this questionnaire. This questionnaire will take 5-10 minutes of your time to complete. All results will be used for research purposes. Be assured that all responses you contribute will be held in the strictest confidentiality. This research is approved by Imam Abdulrahman Bin Faisal University Institutional Review Board Committee.

**Table 5:** The general characteristics of the participants (Part I)

Age in years	( years)
Gender	a. Male ( ) b. Female ( )
Nationality	a. Saudi b. Other nationality ( )
Faculty designation	Biomedical ( ) b. Clinical ( )
Specialty	( )
Degree of qualification	Professor ( ) b. Associate Prof. ( ) c. Assistant Prof. ( ) d. lecturer ( ) e. Consultant ( ) f. Specialist ( ) g. Others specify ( )
Years of experience	Less than 5 years 5- 10 years Over 10 years
Native language	( )
Have you received any training on applying formative assessment in the form of workshop/seminar/webinar?	Yes b. ( )
Do you usually use informal formative assessment (class non-scored assessment) to evaluate your students?	a. Yes b. ( )
Do you usually use formal formative assessment (non-scored assignment as Quizzes/ post rotation exam, surveys, and questionnaires) to evaluate your students?	a. Yes b. ( )

**Table 6:** Formative assessment tool (Part II)

1- Functions of formative evaluation and understanding of its usefulness (4 items)							
Item	Strongly agree (5)	Agree (4)			Unsure (3)	Disagree (2)	Strongly disagree (1)
Identifying the student's strengths and weaknesses							
Guiding student progress							
Increasing the student's autonomy							
Direct teaching plan							
2- Sharing and addressing educational attention and success criteria (5 items)							
Item	Strongly agree (5)	Agree (4)			Unsure (3)	Disagree (2)	Strongly disagree (1)
I share the objectives of each course with my students							
I explain the learning goals to achieve with my students							
I discuss the criteria for success with my students							
I discuss the modalities of the summative assessment with my students							
I discuss the modalities of formative assessment with my students							
3- Methods of performing the formative evaluation (6 items)							
Item	Strongly agree (5)	Agree (4)		Unsure (3)		Disagree (2)	Strongly disagree (1)
Classroom questioning							
Exercises and tests							
Group discussion							
Self-evaluation							
Peer evaluation							
Digital assessment							
4- Temporality of formative evaluation and guidance (11 items)							
Item	Strongly agree (5)	Agree (4)		Unsure (3)		Disagree (2)	Strongly disagree (1)
I do the formative assessment after each teaching activity							
I do the formative assessment at the end of a session of the course							
I do the formative assessment at the end of a course							
I do the formative assessment before the summary evaluation of a course							
I give formative feedback to my students after the formative evaluation activities							
I give individual feedback after the activities of the formative evaluation							
I propose a regulation such as ‘remaking one or more sequences of teaching’							
I propose a regulation such as ‘change the teaching material’							
I propose a regulation such as ‘increase the hourly volume of a course’							
I propose a regulation such as ‘give more explanations’							
I propose a regulation such as ‘do additional exercises’							
5- Instructor’s training (6 items)							
Item	Strongly agree (5)	Agree (4)		Unsure (3)		Disagree (2)	Strongly disagree (1)
My initial training is not sufficient to practice the formative assessment							
Ongoing training on formative assessment will be useful to me							
I expect further training in theoretical contributions to this practice							
I expect training to work on the practicalities of this practice							
I expect continuous training to provide regulatory procedures							
I expect continuous training on how to make a digital formative assessment							
6- Obstacles to the application of formative evaluation (5 items)							
Item	Strongly agree (5)	Agree (4)	Unsure (3)			Disagree (2)	Strongly disagree (1)
Lack of support from the administration to use formative assessment							
Number of students in class							
Activities required for the teacher							
Time required for formative assessment activities							
Student engagement							

## References

[1] Schuwirth LW, Van der Vleuten CP (2018). How to design a useful test: the principles of assessment. Understanding Medical Education: Evidence, Theory, and Practice, Third Edition.

[2] Hattie J (2012). Visible Learning for Teachers: Maximizing Impact on Learning, 1st Edition.

[3] Rudolph JW, Simon R, Raemer DB, Eppich WJ (2008). Debriefing as formative assessment: closing performance gaps in medical education. Acad Emerg Med.

[4] Moore DE (2018). Assessment of learning and program evaluation in health professions education programs. New Directions for Adults and Continuing Education.

[5] Lee H, Chung HQ, Zhang Y, Abedi J, Warschauer M (2020). The effectiveness and features of formative assessment in US K-12 education: a systematic review. Appl Meas Educ.

[6] Allal L, Lopez LM (2005). Formative assessment of learning: a review of publications in French. Formative Assessment: Improving Learning in Secondary Classrooms.

[7] William D (2014). Formative assessment and contingency in the regulation of learning processes. Toward a Theory of Classroom Assessment as the Regulation of Learning.

[8] Swanwick T (2013). Understanding Medical Education: Evidence, Theory, and Practice, 2nd Edition. https://www.wiley.com/en-be/Understanding+Medical+Education%3A+Evidence%2C+Theory+and+Practice%2C+2nd+Edition-p-9781118472361.

[9] Black P, Wiliam D (2018). Classroom assessment and pedagogy. Assess Educ.

[10] Palmen LN, Vorstenbosch MA, Tanck E, Kooloos JG (2015). What is more effective: a daily or a weekly formative test?. Perspect Med Educ.

[11] Raupach T, Schuelper N (2018). Reconsidering the role of assessments in undergraduate medical education. Med Educ.

[12] Gentile M (2020). Understanding the Importance of Formative Assessment Programs in Undergraduate Medical Education. https://core.ac.uk/download/pdf/346296922.pdf.

[13] Abu Musa M, Islam MR (2020). The problems that teachers face in applying formative assessment in the classroom. IJSTR.

[14] McManus S (2008). Attributes of Effective Formative Assessment. https://www.ccsso.org/sites/default/files/2017-12/Attributes_of_Effective_2008.pdf.

[15] Al-Wassia R, Hamed O, Al-Wassia H, Alafari R, Jamjoom R (2015). Cultural challenges to implementation of formative assessment in Saudi Arabia: an exploratory study. Med Teach.

[16] Alsubaiai HS (2021). Teachers’ perception towards formative assessment in Saudi universities’ context: a review of literature. Engl Lang Teach.

[17] Wang X (2007). A case study of transition from summative to formative assessment in Chinese context. Proceedings of the World Congress on Engineering and Computer Science 2007.

[18] Abraham RM, Singaram VS (2016). Third-year medical students’ and clinical teachers’ perceptions of formative assessment feedback in the simulated clinical setting. Afr J Health Prof Educ.

[19] Biswas S, Bhar A, Adhikari A (2021). Continuous formative assessment in teaching-learning anatomy in a medical college of West Bengal: perception of students & teachers. IJBAMR.

[20] Dolin J, Black P, Harlen W, Tiberghien A (2018). Exploring relations between formative and summative assessment. Transforming Assessment: Through an Interplay Between Practice, Research and Policy.

[21] Baig M, Gazzaz ZJ, Farouq M (2020). Blended learning: the impact of blackboard formative assessment on the final marks and students’ perception of its effectiveness. Pak J Med Sci.

[22] Almuntasheri S (2016). Saudi teachers’ practices of formative assessment: a qualitative study. PEC.

[23] Norcini J, Anderson MB, Bollela V (2018). 2018 consensus framework for good assessment. Med Teach.

[24] Lajane H, Gouifrane R, Qaisar R, Chemsi G, Radid M (2020). Perceptions, practices, and challenges of formative assessment in initial nursing education. The Open Nursing Journal.

[25] Konopasek L, Norcini J, Krupat E (2016). Focusing on the formative: building an assessment system aimed at student growth and development. Acad Med.

[26] Chang EK, Wimmers PF (2017). Effect of repeated/spaced formative assessments on medical school final exam performance. Health Prof Educ.

[27] Aslantas I (2016). Teachers’ Perceptions of Using Formative Assessment Methods in the Classroom. https://www.researchgate.net/publication/316889769_Teachers'_perceptions_of_using_formative_assessment_methods_in_the_classroom.

[28] Mulliner E, Tucker M (2017). Feedback on feedback practice: perceptions of students and academics. Assess Eval High Educ.

[29] Cobb KA, Brown G, Jaarsma DA, Hammond RA (2013). The educational impact of assessment: a comparison of DOPS and MCQs. Med Teach.

[30] Bok HG, Teunissen PW, Favier RP (2013). Programmatic assessment of competency-based workplace learning: when theory meets practice. BMC Med Educ.

[31] Khapre MP, Sabane H, Singh S, Katyal R, Kapoor A, Badyal DK (2020). Faculty's perspective on skill assessment in undergraduate medical education: qualitative online forum study. J Educ Health Promot.

[32] Goswami S, Sahai M (2015). Problems and challenges in medical education in India. Eur J Contemp Educ.

[33] Yorke M (2003). Formative assessment in higher education: moves towards theory and the enhancement of pedagogic practice. High Educ.

[34] Do Quyen NT, Khairani AZ (2017). Reviewing the challenges of implementing formative assessment in Asia: the need for a professional development program. J Soc Sci.

[35] Alotaibi KA (2019). Teachers’ perceptions on factors influence adoption of formative assessment. JEL.

